# Effect of Silane Surface Treatments on the Interfacial Shear Strength Between Cotton Yarn and Poly(Lactic Acid) Resin

**DOI:** 10.3390/ma18194582

**Published:** 2025-10-02

**Authors:** Gyu Hyeon Kim, Young Soo Cho, Gye Hwa Shin, Jun Tae Kim

**Affiliations:** 1 Department of Food Science and Technology, Keimyung University, Daegu 42601, Republic of Korea; khyun3259@hanmail.net; 2 Department of Food and Nutrition, Kyung Hee University, Seoul 02447, Republic of Korea; dudtn6245@naver.com; 3 Department of Food and Nutrition, Kunsan National University, Gunsan 54150, Republic of Korea; 4 BioNanocomposite Research Center, Kyung Hee University, Seoul 02447, Republic of Korea

**Keywords:** interfacial shear strength, cotton yarn, poly(lactic acid), silane, surface modification, composites

## Abstract

This study explores the enhancement of mechanical properties in cotton yarn-reinforced poly(lactic acid) (PLA) biocomposites, aimed at providing a sustainable alternative to petroleum-based plastics. The primary challenge addressed is the low interfacial shear strength (ISFF) between the hydrophilic cotton yarn and the hydrophobic PLA matrix. To overcome this, cotton yarn surface was chemically modified using silane treatment. Cotton yarns were aligned on a metal frame and treated with hydrolyzed silane solutions at concentrations of 1%, 2%, 3%, and 4% (*w*/*v*) for 3 h. Although the tensile stress of the cotton yarn decreased significantly (*p* < 0.05) with higher silane concentrations, from 520.46 MPa (untreated) to 340.88 MPa (4% silane-treated), the IFSS improved significantly (*p* < 0.05) from 5.63 MPa to 12.12 MPa. Consequently, the tensile stress of the cotton yarn/PLA biocomposites increased significantly (*p* < 0.05), from 20.74 MPa (untreated) to 41.58 MPa (4% silane-treated). This is because the increased IFSS achieved through silane treatment allowed the PLA polymer to more firmly connect adjacent cotton fibers, resulting in maximum strength. FTIR and SEM analyses confirmed successful surface modification of the cotton yarn. These findings demonstrate that silane treatment effectively enhances interfacial bonding between cotton yarn and PLA resin, leading to improved mechanical performance of the biocomposites.

## 1. Introduction

Petroleum-based plastics are widely used across nearly all industries and human activities due to their excellent mechanical properties, ease of processing, and low cost. However, their non-biodegradable and non-renewable nature has led to significant atmospheric, soil, and water pollution [1,2]. As a result, environmental awareness and the growing scarcity of fossil resources have driven a surge in research on biodegradable plastics and plant-based fiber-reinforced biocomposites over recent decades [2,3]. In particular, plant-derived fibers such as hemp, jute, ramie, coir, sisal, flax, and cotton have been incorporated with biopolymer resins, such as poly(lactic acid) (PLA), soy proteins, and polysaccharides, to produce fiber-reinforced composites [4,5].

PLA has demonstrated comparable or superior barrier, mechanical, and physical properties to those of petroleum-based polymers. For example, the tensile strength and flexural modulus of PLA are higher than those of low-density polyethylene (LDPE), high-density polyethylene (HDPE), polypropylene (PP), and polystyrene (PS). Furthermore, PLA exhibits lower permeabilities to H_2_O, O_2_, CO_2_, and N_2_ gas compared to PS [6,7,8,9,10]. PLA is also biodegradable and compostable, derived from sustainable resources (mainly corn starch), and shows better moisture resistance than other biodegradable polymers. Due to these properties, PLA is considered a promising alternative for waste reduction. Its low environmental toxicity and high processability make it an ideal material for food packaging and other consumer products [11,12,13].

Plant-based fibers offer a completely sustainable, renewable, and biodegradable resource [14]. When used as reinforcing agents in composites, these fibers are advantageous in applications such as thermal insulation, soundproofing, and the automobile industry, owing to their low cost, low density, good thermal properties, and reduced abrasion [15,16]. However, a major drawback lies in the incompatibility between hydrophilic natural cellulose fibers and hydrophobic polymer matrices during composite processing [16].

The fiber–matrix interface plays a crucial role in the mechanical performance of fiber-reinforced composites. A weak interface leads to poor stress transfer, resulting in materials that are generally weak, stiff, and susceptible to fracture. On the other hand, a strong interface enhances both strength and stiffness but may reduce fracture resistance [17]. A poor interfacial bond reduces the contribution of the fibers to stress transfer, weakening the overall composite and shortening its lifespan [18]. Therefore, several experimental techniques have been developed to evaluate interfacial bonding and characterize interfacial shear strength (IFSS). The pull-out test is one of the most widely used methods for such evaluations [17,19,20].

Despite significant progress in developing fiber-reinforced composites, there is a critical gap in understanding how to optimize the interfacial bonding between plant-based cellulose fibers and polymer matrices. Most existing studies have focused on general surface treatments, leaving a lack of comprehensive investigations into specific chemical modifications, such as silane treatment, and their systematic optimization. Particularly underexplored is the impact of varying silane concentrations on the interfacial shear strength and mechanical performance of cotton yarn/PLA biocomposites. Addressing this gap is essential to advancing the development of high-performance, sustainable composite materials that can meet industrial demands.

Since strong interfacial interactions improve composite strength, modifying the surface of hydrophilic plant-based cellulose fibers to become hydrophobic is often necessary. Fibers such as hemp, jute, ramie, coir, sisal, flax, and cotton naturally possess hydrophilic surfaces due to their abundant hydroxyl (-OH) groups. Surface treatments with silane, organic and inorganic acids, sodium hydroxide, or peroxides can introduce functional groups that enhance bonding between fibers and the polymer matrix [17]. Among these, silane coupling agents are particularly effective. These compounds offer three main advantages: (1) they are suitable for large-scale industrial applications; (2) they possess alkoxysilane groups that readily react with the hydroxyl groups on cellulose fiber surfaces; and (3) they carry various functional groups that improve compatibility and enable covalent bonding between the fiber and matrix [21,22].

This study hypothesizes that chemically modifying the cotton yarn surface with silane treatment will enhance the IFSS between the cotton yarn and the PLA matrix, thereby improving the overall mechanical properties of the cotton yarn/PLA biocomposites. The modified cotton yarn was characterized using liquid wicking rate measurements and FT-IR spectroscopy. In addition, the impact of silane concentration on IFSS and the tensile properties of the resulting cotton yarn-reinforced biocomposites was systematically investigated.

## 2. Materials and Methods

### 2.1. Materials

Cotton yarn was purchased from a local market in Daegu, Republic of Korea. 3-(2-aminoethlyamino)propyltrimethoxysilane and polyethylene glycol (PEG 600) were purchased from Sigma Aldrich Co. (St. Louis, MO, USA). Poly(lactic acid) (PLA 2003D) was purchased from Green Chemical Co., Ltd. (Incheon, Republic of Korea). Chloroform (>99.5%) and acetic acid were obtained from Samchun Chemical (Yeosu, Republic of Korea) and Kanto Chemical Co., Inc. (Tokyo, Japan), respectively.

### 2.2. Surface Modification of Cotton Yarn

**Figure 1:**
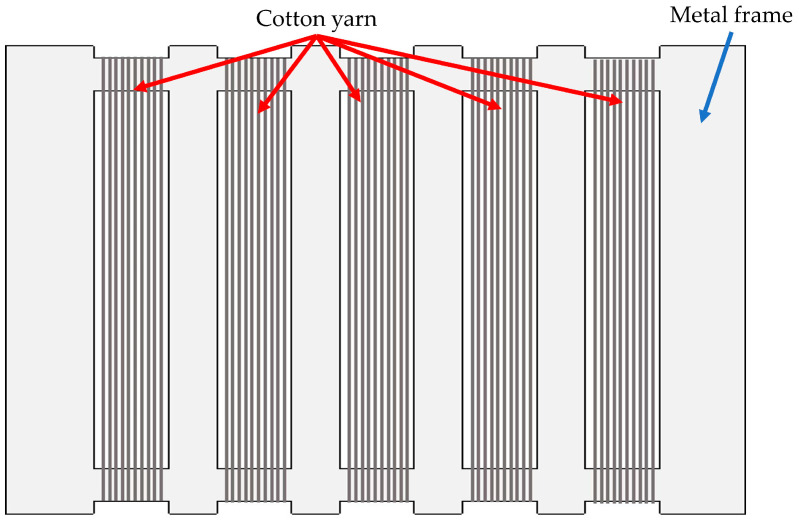
A schematic of aligned-cotton yarn wound on a metal frame.

**Figure 2:**
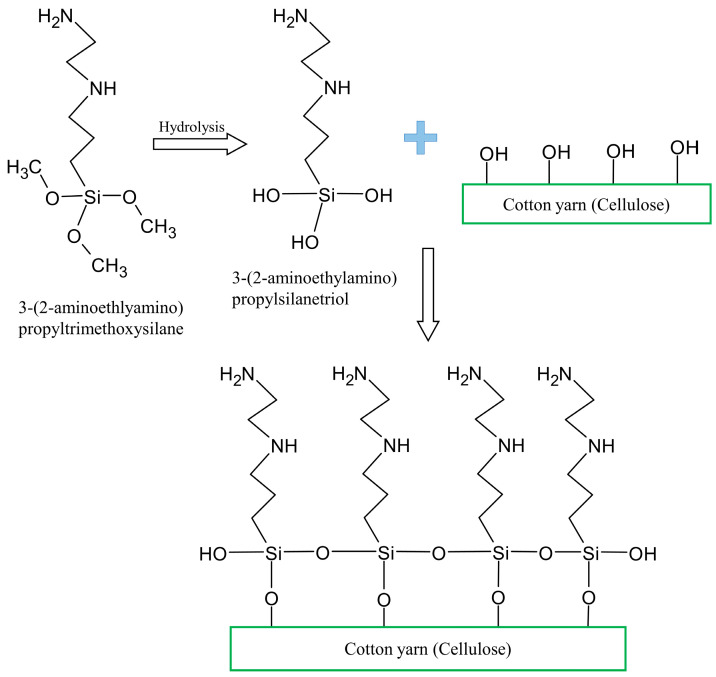
Schematic diagram of the chemical modification of cotton yarn cellulose by silane treatment.

The surface of cotton yarn was treated with 3-(2-aminoethlyamino)propyltrimethoxy -silane as a coupling agent. First, 1%, 2%, 3%, and 4% (*w*/*v*) silane solutions were hydrolyzed in a mixture of ethanol and distilled water (60/40, *v*/*v*) under magnetic stirring for 1 h. Cotton yarns were tightly wound onto a metal frame, as shown in Figure 1, to minimize the loosening of the fiber alignment. The whole frame with cotton yarns was immersed in the hydrolyzed silane solution (3-(2-aminoethlyamino)propylsilanetriol) at room temperature (RT) for 3 h. Then, the pH of the solution was adjusted to 4 using 99.7% acetic acid, and the cotton yarn was washed with distilled water until the pH became neutral. The wet yarn was dried in an oven at 100 °C for 12 h and then stored in a constant temperature and humidity chamber at 21 °C and 65% relative humidity (RH) before characterization. Chemical modification of cotton yarn cellulose surface by silane treatment was shown in Figure 2.

### 2.3. Analysis of Cotton Yarn

#### 2.3.1. Fourier-Transform Infrared Spectroscopy (FTIR)

Attenuated total reflection-Fourier-transform infrared (ATR-FTIR) spectroscopy (Thermo SCIENTIFIC, ATR iD5, Nicolet iS5, Waltham, MA, USA) was used to analyze the characteristic functional groups of pure and silane-modified cotton yarns. A 5 mm diameter disc-shaped cotton yarn sample was used. All IR spectra were measured in transmission mode in the range of 3600–900 cm^−1^ with a resolution of 4 cm^−1^ and a scan rate of 16 scans/min.

#### 2.3.2. Optical Microscopy

Optical microscopy (OLYMPUS BX43F, Tokyo, Japan) was used to characterize the surface of pure and silane-modified cotton yarns. The yarn diameter, twist direction, and twist angle were measured before and after modification, and the results were compared.

#### 2.3.3. Tensile Properties of Cotton Yarn

**Figure 3:**
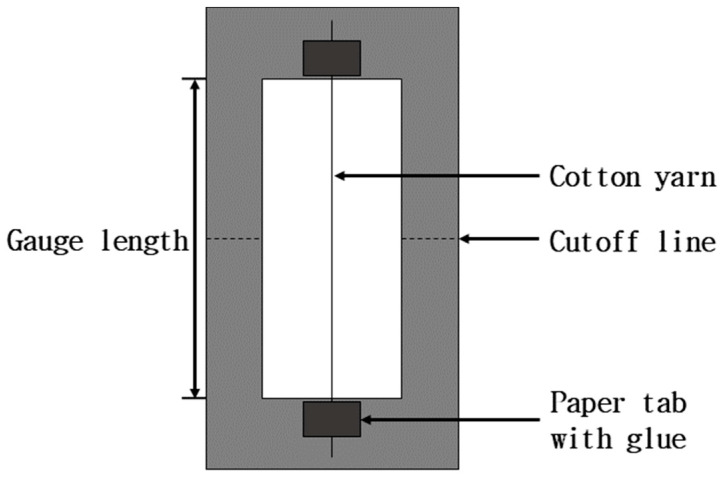
A schematic of a single cotton yarn attached to a paper tab for tensile test (Adapted with permission from [14]. Copyright © 2010 Elsevier Ltd.).

The tensile properties of a single cotton yarn were measured using a Universal Testing Machine with 100 N load (UTM; Zwick Z010TN, Zwick GmbH & Co., KG, Ulm, Germany). Figure 3 shows the schematic of the cotton yarn specimen used for the tensile test. The cotton yarn was individually attached to a rectangular paper frame using instant glue. The cross-sectional area was measured to calculate the tensile stress of the yarn. However, since the yarn had an irregular diameter and non-circular cross-section, a more accurate cross-sectional area was obtained by the following equation:

$$Area\;\left(m^{2}\right)=\;\frac{Linear\;density\;of\;yarn\;(g/1000\;m)}{Fiber\;density\;(g/{cm}^{3})}$$

The average linear density of the yarn was determined by weighing ten 100 cm specimens, which was 27.72 tex (g/1000 m). The cross-sectional area was calculated using the cotton fiber density (1.5–1.6 g/cm^3^) [23]. All yarn specimens were conditioned at 21 °C and 65% RH for 24 h before testing. The gauge length was 50 mm, and the strain rate was 0.2 mm/min. The cutoff line was cut immediately before testing the yarns. At least 20 specimens were tested to obtain average tensile properties.

#### 2.3.4. Liquid Wicking Rate

The liquid wicking rate of untreated and silane-treated cotton yarns was measured according to the DIN 53924 protocol (a test based on the hydrophilic behavior of yarns) [24] with slight modifications. One end of the cotton yarn was immersed in colored distilled water, and the liquid was allowed to move along the yarn by capillary action. The water absorption rate of the yarns and the distance traveled by the liquid were measured using a caliper with an accuracy of 0.01 mm (Absolute Digimatic Calipers CD-15CPX, Mitutoyo, Kawasaki, Japan). The height of the rising water interface was recorded after 5 min, and the measurements were repeated 10 times for each sample [25,26].

### 2.4. Preparation of Cotton Yarn/PLA Biocomposites

PLA resin was prepared by dissolving PLA pellets in 10 times volume of chloroform with 10% PEG 600 (*w*/*w*, based on PLA) under stirring overnight. When the PLA was completely dissolved, the solution was left at room temperature until all bubbles disappeared. To prepare cotton yarn-reinforced PLA biocomposites, 110 mm-long cotton yarn was wound 10 times around a metal frame. The metal frame wound with cotton yarn was placed on a Teflon-coated plate, and 50 mL of PLA resin was poured over the cotton yarn. The composites were prepared by drying the cotton yarn in an exhaust hood at RT for 12 h to evaporate all chloroform. Dried composites were cured at 60 °C for 15 min under 5 MPa. The cured composites were conditioned at 21 °C and 65% RH for 48 h before characterization.

### 2.5. Analysis of Cotton Yarn/PLA Biocomposites

#### 2.5.1. Interfacial Shear Strength (IFSS) Between Cotton Yarn and PLA Resin

**Figure 4:**
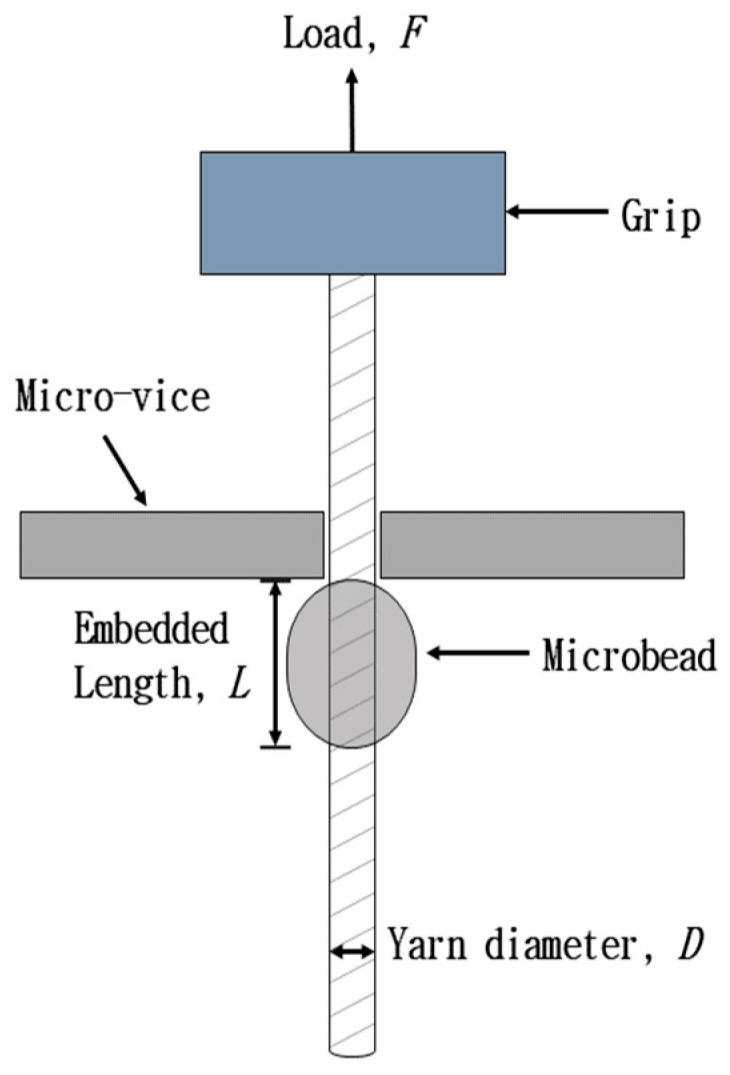
A schematic of the pull-out test for measuring the interfacial shear strength between cotton yarn and PLA resin (Adapted with permission from [5]. Copyright © 2011 Elsevier Ltd.).

Interfacial shear strength (IFSS) of cotton yarn/PLA biocomposites was characterized using a pull-out test [3,5]. As a pre-treatment, a small microbead was mounted onto the cotton yarn using the PLA resin. The samples were stored in a drying oven at 25 °C for 12 h to evaporate the remaining chloroform. All samples were equilibrated at 21 °C and 65% RH for 24 h before performing the pull-out test. Figure 4 shows a schematic diagram of the pull-out test to measure the IFSS between the cotton yarn and PLA resin. The embedded length (L) and fiber diameter (D) were measured using an optical microscope. The surface area was calculated using these values. The pull-out test was performed using a specially manufactured micro-vise clamp equipped with a universal testing machine (UTM). The flat micro-vise plates were placed above the microbead and brought close to the cotton yarn until it almost touched the surface. The cotton yarn was then pulled out from the microbead at a test speed of 0.2 mm/min until the microbead detached. The IFSS (τ) was calculated using the following equation:

$$Interfacial\;shear\;strength\;(\tau )=\frac{F}{\pi \times D\times L}$$

where F is the force required to detach the microbead. At least 20 tests were conducted to obtain the average IFSS values.

#### 2.5.2. Tensile Properties of Cotton Yarn/PLA Biocomposites

The tensile properties (Young’s modulus, tensile stress, and tensile strain) of cotton yarn/PLA biocomposites were measured using a UTM according to ASTM D882-10 [27]. The dried composites were cut into rectangular strips of 100 mm × 10 mm and then conditioned at 21 °C and 65% RH for 48 h. The thickness of each specimen was measured at five random points using a digital micrometer (Mitutoyo Co., Kanagawa, Japan) to the nearest 0.001 mm. The composites were stretched at a cross-head speed of 2 mm/min using a UTM with a 100 N load cell. The theoretical Young’s modulus and tensile stress of the cotton yarn/PLA biocomposites were calculated using the following equation:

$$Theoretical\;value=(T_{y}\times V_{y})+(T_{r}\times V_{r})$$

where T_y_ and T_r_ represent the tensile properties of yarn and resin, respectively, while V_y_ and V_r_ represent the volume fractions of yarn and resin, respectively. The theoretical and experimental values were compared. At least 10 specimens were tested to obtain average tensile properties of the composites.

#### 2.5.3. Scanning Electron Microscopy (SEM)

The surface and fracture surface of the cotton yarn/PLA biocomposites were analyzed using a scanning electron microscope (SEM; S-4800 FE-SEM, Hitachi Ltd., Tokyo, Japan). The specimens were mounted on a specimen holder using carbon tape and sputtered with a thin layer of osmium for 5 s using an osmium plasma coater (OPC-60A, West Chester, PA, USA). The coated specimens were observed using an acceleration voltage of 15 kV.

### 2.6. Statistical Analysis

All experiments were performed on conditioned samples, with results reported as mean and standard deviation (mean ± SD), based on three replicates for each condition to ensure reliability. Statistical analysis was conducted using one-way analysis of variance (ANOVA) with IBM SPSS software (Version 25, SPSS Inc., Chicago, IL, USA) to assess variance among groups. Duncan’s multiple range test was applied to detect significant differences between sample means, with a significance level set at *p* < 0.05. This approach ensured robust error analysis and highlighted variations across treatments.

## 3. Results and Discussion

### 3.1. Characterization of Cotton Yarn

#### 3.1.1. Effect of Silane Surface Treatment on FTIR Spectra of Cotton Yarn

**Figure 5:**
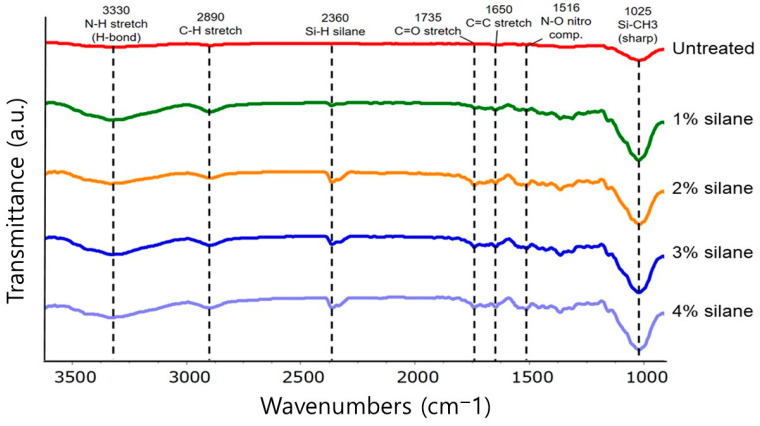
FTIR spectra of the untreated cotton yarn and cotton yarns treated with various concentrations of silane.

FTIR spectra of untreated cotton yarn (control) and cotton yarn treated with various concentrations of silane are shown in Figure 5. Untreated cotton yarn showed a broad band at 1025 cm^−1^, whereas silane-treated cotton yarns showed sharper bands with increasing silane concentration. These sharper bands correspond to the Si-OR bond of Si in silane and the hydroxyl groups (-OH) present on the cotton yarn surface. The typical absorption peaks of the Si-O-Si bond of siloxane compounds in the region of 1000–1130 cm^−1^ appear to overlap with the cellulose band due to C-O bending [28]. In addition, the bands between 2100 and 2360 cm^−1^ correspond to the Si-H bond of silane. The amine group band of silane provides important evidence for the amino-silane bond on the treated cotton yarn surface [29]. For example, the bands in the range of 1470–1650 cm^−1^ are associated with the symmetric and asymmetric deformation vibrations of protonated amines. The band corresponding to the N-H stretching vibration was observed at 3000–3500 cm^−1^ [29]. The IR spectral differences between the untreated and silane-treated cotton yarns provide compelling evidence of the successful grafting of silane onto the cotton surface. This grafting not only modified the surface chemistry but also enhanced the material properties, potentially improving durability, hydrophobicity, and compatibility with hydrophobic polymers such as PLA. Analyzing the FTIR spectra shows that silane treatment effectively altered the cotton yarn’s surface, offering a pathway to advanced material applications.

#### 3.1.2. Effect of Silane Surface Treatment on Yarn Diameter and Twist Angle of Cotton Yarns

**Figure 6:**
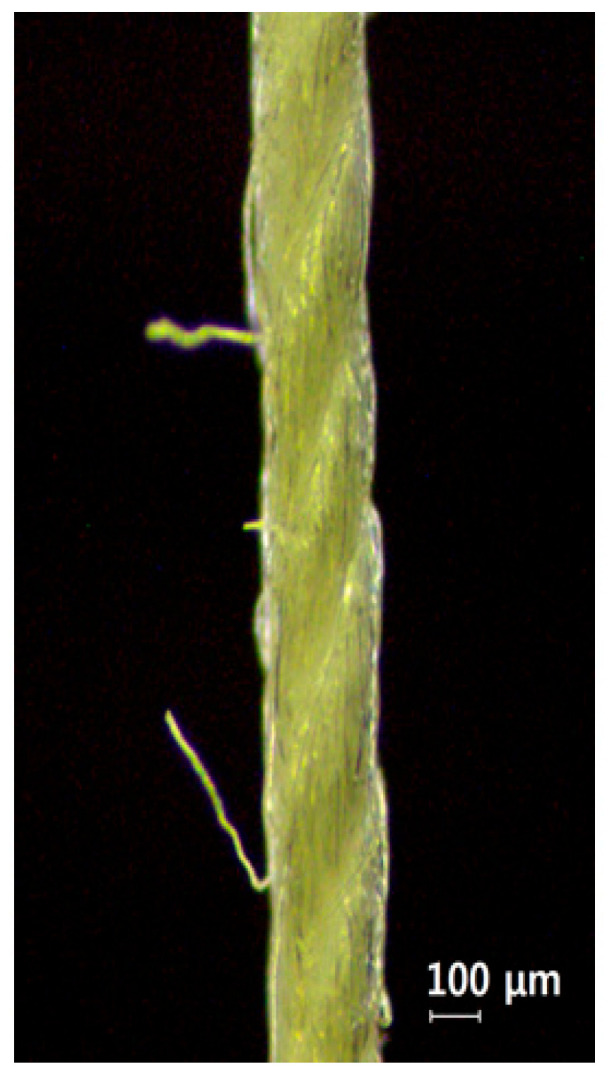
Microscopic image of a single cotton yarn. Imaging was performed at magnification of 40×.

The yarn diameter, twist direction, twist angle, and the microbeads on the cotton yarn for the IFSS test were observed using an optical microscope. Table 1 shows the yarn diameter and twist angle before and after silane modification of cotton yarn. The diameter of untreated cotton yarn was 186.6 μm and twist angle was 27.3°. After silane treatment, both the diameter and the twist angle of cotton yarn significantly (*p* < 0.05) decreased, but there was no significant difference among the various silane concentrations. The diameter and twist angle of the cotton yarn modified with 4% silane were 173.5 μm and 22.5°, respectively. Figure 6 shows longitudinal image of the untreated cotton yarn, taken using an optical microscope. The cotton fibers were twisted in the right-handed direction (Z-twist) and some microfibers appeared to be protruding out of the yarn because it was formed by twisting many short fibers [5].

#### 3.1.3. Effect of Silane Surface Treatment on Tensile Properties of Cotton Yarns

**Figure 7:**
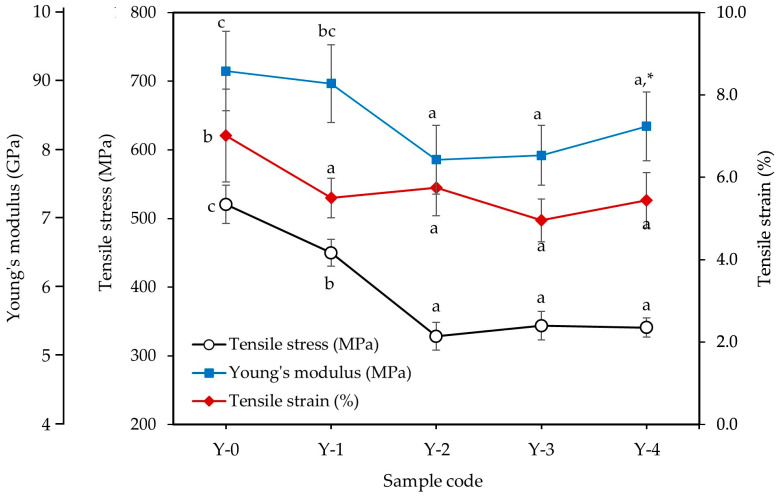
Effect of silane concentrations on the tensile properties of cotton yarn. * Different letters in the same variant indicate a significant difference at *p* < 0.05 by Duncan’s multiple range test.

The effect of silane treatment on the tensile properties of cotton yarns is shown in Figure 7. The Young modulus, tensile stress, and tensile strain of untreated cotton yarn were 8.58 GPa, 520.46 MPa, and 7.01%, respectively. These values are consistent with those reported in other studies [16,23]. Silane-treated cotton yarns had significantly (*p* < 0.05) lower tensile properties than untreated cotton yarns. The tensile stress of the yarn treated with 1% silane was 449.92 MPa, and that of the yarn treated with 4% silane was 340.88 MPa. Similarly, the Young modulus decreased from 8.27 GPa for the yarn treated with 1% silane to 7.24 GPa for the yarn treated with 4% silane. The average tensile strain of the silane-treated yarns was 5.4%. The mechanical properties of cotton yarn are related to the microstructure of cotton fibers and the possibility of damage and cleavage of β-1,4-glucosidic bonds in the cellulose structure of cotton fibers during silane treatment [23]. Similar results were reported in the sisal fibers modified by silane and NaOH solution [3].

#### 3.1.4. Effect of Silane Surface Treatment on the Liquid Wicking Rate of Cotton Yarns

**Figure 8:**
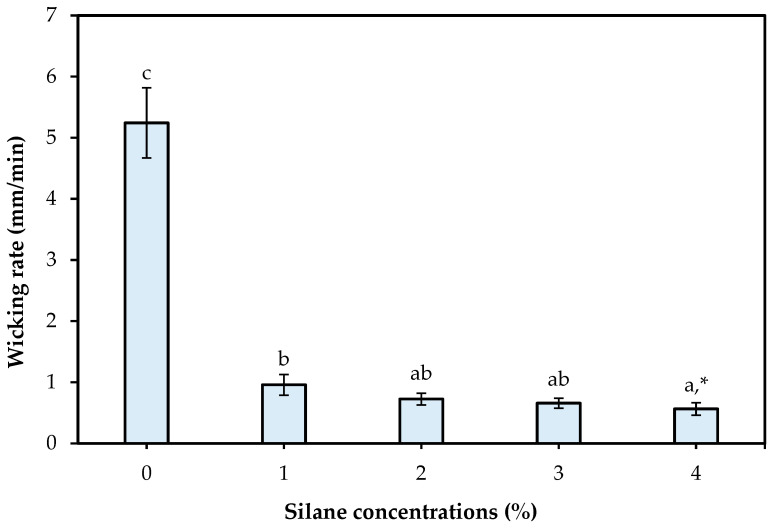
Effect of silane concentrations on the liquid wicking rate of silane treated cotton yarn. * Different letters indicate a significant difference at *p* < 0.05 by Duncan’s multiple range test.

The liquid wicking test was performed to confirm if the silane-treated cotton yarn was hydrophobic. Previous studies have shown that silane treatment can impart hydrophobicity to the surface of cellulose fibers [3,28,30]. As shown in Figure 8, the wicking rate of untreated cotton yarn was 5.24 mm/min, whereas the wicking rates of the cotton yarns treated with 1%, 2%, 3%, and 4% silane significantly (*p* < 0.05) decreased to 0.96, 0.73, 0.66, and 0.56 mm/min, respectively. However, no significant (*p* > 0.05) difference in the wicking rate was observed in yarns treated with silane concentrations higher than 2%. Liquid wicking occurs because of fiber–liquid interactions, such as capillary penetration, fiber moisture absorption, and intrafiber liquid diffusion [31,32]. A lower liquid wicking rate indicates less active fiber–liquid interactions, indicating higher hydrophobicity. These results suggest that silane treatment imparted hydrophobicity to cotton yarns, which could form strong bonds with the hydrophobic PLA resin.

### 3.2. Characterization of Cotton Yarn/PLA Biocomposites

#### 3.2.1. Effect of Silane Surface Treatment on FTIR Spectra of Cotton Yarn

**Figure 9:**
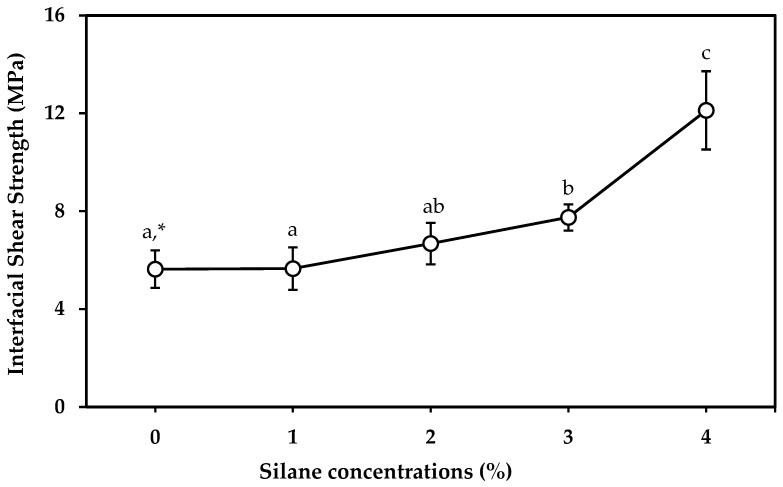
Effect of silane concentrations on the interfacial shear strength (IFSS) between cotton yarn and PLA resin. * Different letters indicate a significant difference at *p* < 0.05 by Duncan’s multiple range test.

Figure 9 shows the IFSS between PLA resin and cotton yarns treated with various silane concentrations. The IFSS of cotton yarn/PLA increased with increasing silane concentration. The IFSS of untreated cotton yarn and 1% silane-treated sample were 5.63 and 5.65 MPa, respectively, with no significant (*p* < 0.05) difference. However, the IFSS of cotton yarn treated with 3% and 4% silane significantly (*p* < 0.05) increased to 7.75 and 12.12 MPa, respectively. Previous studies [3,23,33] attributed this improvement to increased mechanical interlocking at the contact surface between the cotton yarn and PLA resin. The silane structure could strengthen the bond between the cotton yarn surface and PLA polymer resin through siloxane (-Si-O-Si-) bridges. During the silane treatment process, the yarn underwent a series of hydrolysis, condensation, and bond-forming reactions. Silanols (-Si-O-) were formed in the presence of moisture and hydrolyzable alkoxy groups. When the silane-treated yarns dried, a reversible condensation reaction occurred. During the condensation process, one end of the silanol reacted with the -OH group of cellulose, and the other end could react with the polymer resin. This reaction provided molecular continuity across the interface and enhanced the adhesion between the yarn and the resin. Consequently, the IFSS significantly (*p* < 0.05) increased by 215% with 4% silane treatment. These results suggest that the silane treatment enhanced the intermolecular interactions between the cotton yarn and PLA resin. Furthermore, FTIR analysis and the liquid wicking test support the successful surface modification of the cotton yarn by silane treatment.

#### 3.2.2. Effect of Silane Treatment on Tensile Properties of Cotton Yarn/PLA Biocomposites

**Figure 10:**
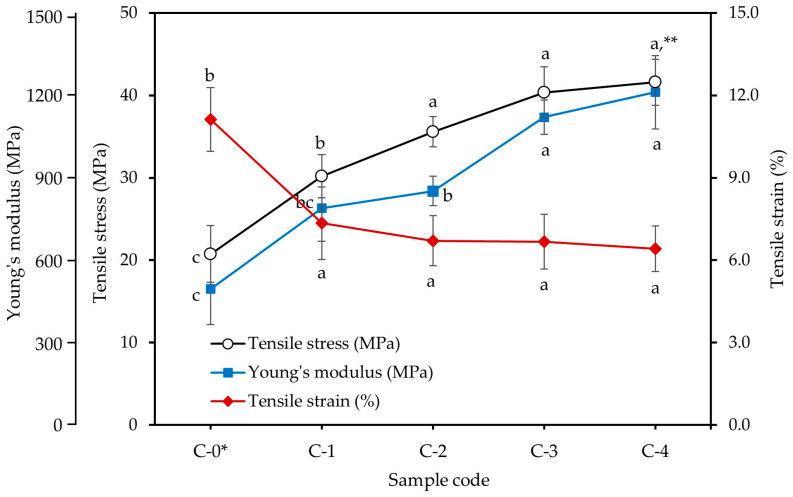
Effect of silane concentrations on the tensile properties of untreated and silane treated cotton yarn/PLA biocomposites. * C-0: untreated cotton yarn/PLA biocomposites, C-1~C-4: silane treated cotton yarn/PLA biocomposites with 1~4% silane concentrations. ** Different letters in the same variable indicate a significant difference at *p* < 0.05 by Duncan’s multiple range test.

The tensile properties of the cotton yarn/PLA biocomposites are shown in Figure 10. The Young modulus, tensile stress, and tensile strain of the untreated cotton yarn/PLA biocomposite were 496.0 MPa, 20.74 MPa, and 11.12%, respectively. After silane treatment, the Young modulus and tensile stress of the biocomposites increased from 788.8 MPa and 30.17 MPa for 1% silane treatment) to 1211.1 MPa and 41.58 MPa for 4% silane treatment, respectively. Therefore, the strength of the biocomposites increased with increasing silane concentration. In particular, the Young modulus and tensile stress increased by 226% and 195% for 3% silane treatment and 244% and 201% for 4% silane treatment, respectively, compared with those of the untreated biocomposite. In contrast, the tensile strain of the silane-treated biocomposites slightly decreased with increasing silane concentrations from 7.34% to 6.42%.

These results differ from the tensile properties of single cotton yarn. Although silane treatment decreased the stiffness of the cotton yarn itself, the stiffness of the biocomposites increased. Table 2 shows the theoretical and experimental values of the tensile properties of cotton yarn/PLA biocomposites. The Young modulus and tensile stress of pure PLA resin were 495.5 MPa and 11.39 MPa, respectively. Untreated cotton yarn/PLA biocomposites showed big differences between theoretical and experimental values. This is because the yarn does not contribute sufficiently to the strength of the biocomposite due to the weak interaction between the cotton yarn and the resin within the biocomposite matrix. However, silane-treated cotton yarn/PLA biocomposites showed a trend toward closer correlation between theoretical and experimental values as silane concentration increased because of the strong interaction between silane-treated cotton yarn and PLA resin. IFSS results also showed increased interaction between silane-treated cotton yarns and PLA resin. In addition, several studies have reported that the properties of fiber-reinforced composites can be improved through silane treatment of fibers [34,35,36].

#### 3.2.3. Effect of Silane Surface Treatment on the Morphology of Cotton Yarn/PLA Biocomposites

**Figure 11:**
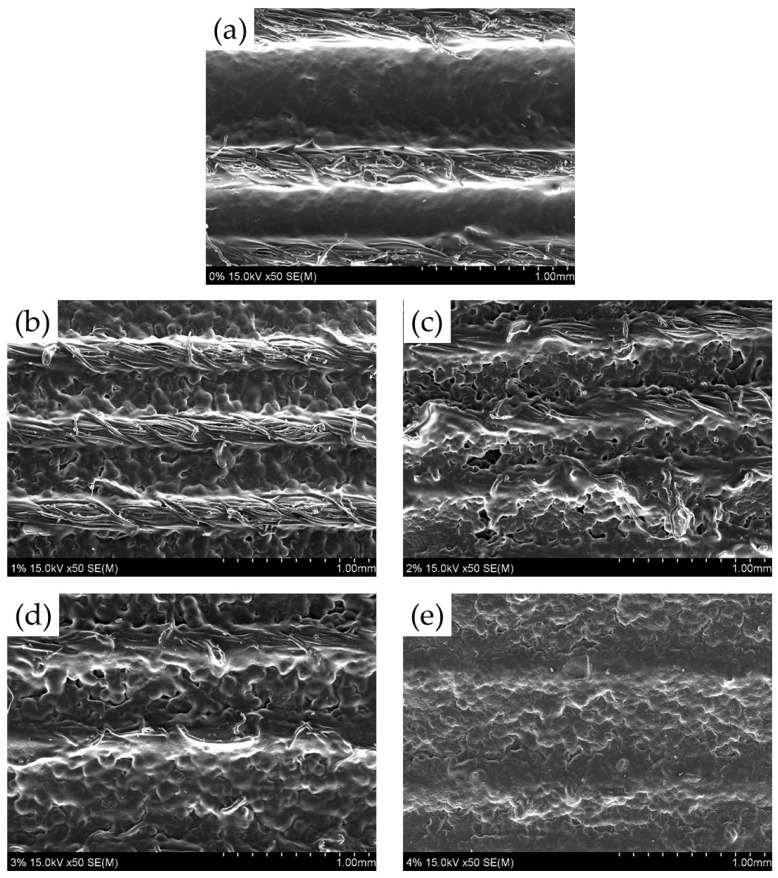
SEM photomicrographs of the surface of cotton yarn/PLA biocomposites. (**a**) Untreated, (**b**) 1% silane treated, (**c**) 2% silane treated, (**d**) 3% silane treated, and (**e**) 4% silane treated cotton yarn/PLA biocomposites.

**Figure 12:**
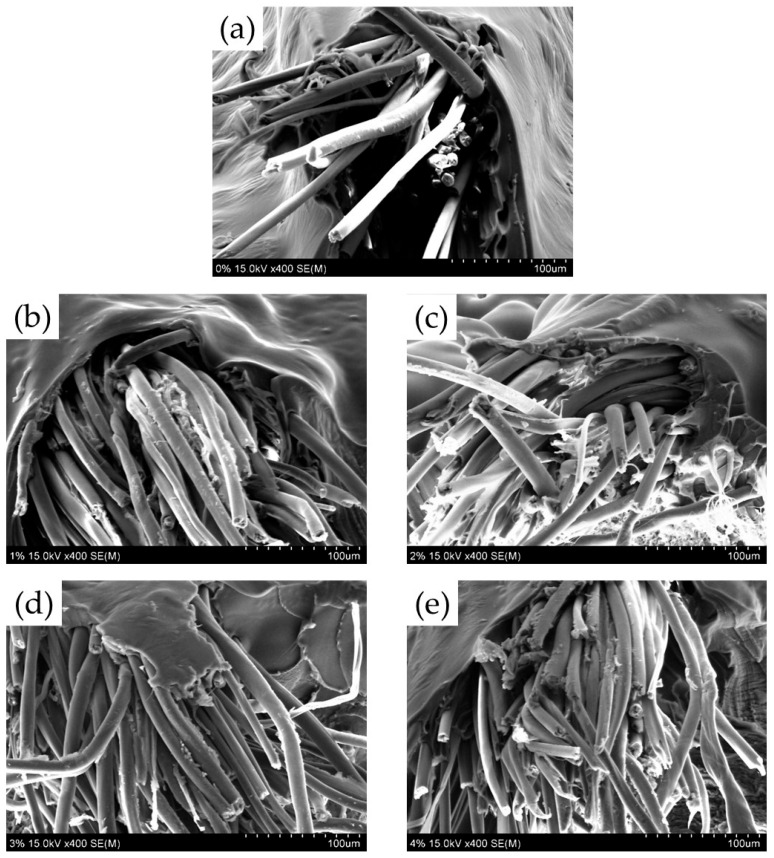
SEM photomicrographs of the fracture surface of cotton yarn/PLA biocomposites. (**a**) Untreated, (**b**) 1% silane treated, (**c**) 2% silane treated, (**d**) 3% silane treated, and (**e**) 4% silane treated cotton yarn/PLA biocomposites.

The surface and fracture surface of cotton yarn/PLA biocomposites were analyzed by SEM. Figure 11 shows the surface of yarn/PLA biocomposites with different silane concentrations. In the untreated and 1% silane-treated cotton yarn/PLA biocomposites, fibers were visible on the surface of the biocomposites because the PLA resin was absent or did not completely coat the yarns. However, the amount of PLA resin on the yarn surface slightly increased in the 2% and 3% silane-treated cotton yarn/PLA biocomposites. The 4% silane-treated cotton yarn/PLA biocomposites did not expose fibers because the PLA resin completely covered the yarns. Furthermore, the surface of the biocomposites became rougher as the silane concentration increased. Figure 12 shows the fracture surfaces of the cotton yarn/PLA biocomposites treated with different silane concentrations. Untreated and 1% silane-treated cotton yarn/PLA biocomposites exhibited gaps between the cotton yarn and the PLA resin, indicating weak interfacial bonding and poor embedding of the cotton yarn within the PLA matrix. In contrast, biocomposites treated with 2%, 3%, and 4% silane showed significantly tighter bonding between the cotton yarn and the PLA resin, with no observable gaps. This improved interface is corroborated by studies demonstrating enhanced interfacial bonding and mechanical performance with increased silane concentration [37]. Specifically, fiber pullout was observed in the untreated biocomposite, creating spaces, whereas fibers in the silane-treated composites adhered more effectively to the yarn surface, forming robust interfaces. These findings align with increased IFSS measurements and enhanced mechanical properties documented in similar research [37].

## 4. Conclusions

Silane modification effectively transformed the surface of cotton yarn from hydrophilic to hydrophobic, as indicated by changes in the liquid wicking rate. Although this modification led to a reduction in the tensile properties of the cotton yarn itself, it significantly enhanced the mechanical properties of the cotton yarn/PLA biocomposites by improvement of IFSS between cotton yarn and PLA resin. For instance, 4% silane-treated cotton yarn/PLA composites exhibited a 244% increase in Young’s modulus and a 201% increase in tensile stress. The interfacial shear strength (IFSS) also improved by 215%, suggesting stronger bonding between the cotton yarn and PLA resin. SEM images confirmed that silane treatment resulted in a more compact structure within the biocomposites, reducing interfacial voids and increasing resin coverage. Theoretical and experimental comparisons highlighted that higher silane concentrations (≥2%) led to better interfacial bonding, improving stress transfer efficiency between the cotton yarn and the PLA matrix. This convergence between experimental and theoretical results underscores the effectiveness of the treatment. Overall, the findings demonstrate that optimized silane surface modification not only enhances the mechanical performance but also increases the reliability of natural fiber-reinforced biocomposites. This improvement positions these materials as promising candidates for commercial applications, offering enhanced durability and structural integrity. The reduction in tensile properties of cotton yarn itself is a limitation that must be considered, especially in applications where the strength of the cotton yarn itself is critical. Furthermore, the cost and environmental impact of using high-concentration silanes must be evaluated to ensure sustainable and economically viable applications.

## Figures and Tables

**Table 1:** Yarn diameter and twist angle of untreated cotton yarn and silane-treated cotton yarns.

	Sample Code				
	Y-0 *	Y-1	Y-2	Y-3	Y-4
Yarn diameter (μm)	186.6 ± 16.9 ^b^	177.9 ± 7.4 ^a^	173.6 ± 6.3 ^a^	175.6 ± 7.7 ^a^	173.5 ± 8.7 ^a^
Twist angle (°)	27.3 ± 3.7 ^c^	23.8 ± 2.5 ^b^	23.1 ± 2.6 ^b^	23.1 ± 2.6 ^b^	22.5 ± 2.5 ^a^

* Y-0: untreated cotton yarn, Y-1~Y-4: silane treated cotton yarn with 1~4% silane concentration. Different letters in the same row indicate a significant difference at *p* < 0.05 by Duncan’s multiple range test.

**Table 2:** Theoretical and experimental tensile properties of cotton yarn/PLA biocomposites.

Samples	Silane Conc.	Volume Fraction of PLA	Young’s Modulus (MPa)		Tensile Stress (MPa)	
			Theoretical	Experimental	Theoretical	Experimental
PLA resin	-	100	-	495.51 ± 67.00	-	11.39 ± 2.53
Cotton yarn/PLA biocomposites	0%	92.96	1064.59	496.01 ± 140.46	47.22	20.74 ± 3.81
	1%	89.06	1346.75	788.77 ± 129.77	59.38	30.19 ± 2.63
	2%	90.10	1082.52	851.76 ± 58.47	42.76	35.53 ± 1.92
	3%	91.45	1012.06	1120.30 ± 52.39	39.81	40.35 ± 3.51
	4%	90.54	1133.48	1211.14 ± 129.63	42.57	41.58 ± 3.22

## Data Availability

The original contributions presented in this study are included in the article. Further inquiries can be directed to the corresponding authors.
